# Difficulties in Schistosomiasis Assessment, Corsica, France

**DOI:** 10.3201/eid2204.160110

**Published:** 2016-04

**Authors:** Hélène Moné, Martha C. Holtfreter, Gabriel Mouahid, Joachim Richter

**Affiliations:** University of Perpignan, Perpignan, France (H. Moné, G. Mouahid); Heinrich-Heine-University Düsseldorf, Düsseldorf, Germany (M.C. Holtfreter, J. Richter)

**Keywords:** schistosomiasis, Schistosoma haematobium, Schistosoma bovis, parasitic disease, helminthiasis, vector-borne infections, zoonoses, zoonotic, Corsica, France, parasites

**To the Editor:** We would like to add some specification and clarification to the discussion regarding the diagnostics and case definitions for urinary schistosomiasis in travelers to Corsica, France (*1*–*3*). Evidence for a *Schistosoma haematobium* infection typically depends on the detection of viable ova in the urine. However, in regard to *S. haematobium* infections acquired in Corsica, several ova excreted by the first 2 published case-patients (i.e., the 12-year-old boy and his father) exhibited atypical morphology (*4*). Therefore, we supplemented our morphologic study with a molecular study of miracidia by using cytochrome c oxydase mitochondrial DNA barcoding and the internal transcribed spacer 2 gene. 

The results indicated that the schistosome responsible for the infection of the first case-patient reported in Corsica was *S. haematobium* that had been introgressed by genes of zoonotic *S. bovis* through a hybridization process. *S. bovis* is the cause of bovine intestinal schistosomiasis and uses the same intermediate host (*Bulinus truncatus *snails) that *S. haematobium* uses (*5*). Such interactions between *S. haematobium* and *S. bovis* have also been reported in Benin (*5*). These findings imply that the clinical course of case-patients and diagnostic test results might be affected by atypical schistosomiasis. Whereas the boy in our study experienced a clinically typical schistosomal infection of the bladder, his father and his siblings, who had identical histories of exposure, were seropositive for *S. haematobium *but were asymptomatic (*4*). 

We recommend that clinicians treat any suspected case of *S. haematobium *infection, whether or not the patient excretes ova, given that the disease is potentially serious and the indicated drug for treatment (praziquantel) is safe. Epidemiologic analyses should take into account the role of zoonotic *S. bovis* infection and supplement parasitological investigations with molecular analyses (*5*,*6*).
